# Electrical Resistance-Based Characterization of Carbon Steel Using Controlled Current Injection and Parameter Estimation

**DOI:** 10.3390/ma19142971

**Published:** 2026-07-10

**Authors:** Gerardo Marx Chávez-Campos, Octavio Vázquez-Gómez, Luis Ulises Chávez-Campos, Antony Morales-Cervantes, Sixtos Antonio Arreola-Villa, Salvador Medina-Alonzo

**Affiliations:** 1 Posgrado en Ciencias en Ingeniería Electrónica, División de Estudios de Posgrado e Investigación, Tecnológico Nacional de México—Instituto Tecnológico de Morelia, Morelia 58120, Michoacán, Mexico; antony.mc@morelia.tecnm.mx (A.M.-C.); m13121122@morelia.tecnm.mx (S.M.-A.); 2 Posgrado en Ciencias en Metalurgia, División de Estudios de Posgrado e Investigación, Tecnológico Nacional de México—Instituto Tecnológico de Morelia, Morelia 58120, Michoacán, Mexico; octavio.vg@morelia.tecnm.mx; 3 Departamento de Ingeniería Electrónica, Tecnológico Nacional de México—Instituto Tecnológico de Morelia, Morelia 58120, Michoacán, Mexico; luis.ulises.cc@morelia.tecnm.mx; 4 Especialidad en Cadena de Valor de Litio, División de Estudios de Posgrado e Investigación, Tecnológico Nacional de México—Instituto Tecnológico de Morelia, Morelia 58120, Michoacán, Mexico; sixtosantonio.av@morelia.tecnm.mx

**Keywords:** electrical resistance, electrical resistivity, carbon-based, least squares, system identification, DC-DC converter, four-points

## Abstract

Electrical resistance measurements can provide supplementary information for the characterization of carbon steels, since resistivity is influenced by composition, microstructure, geometry, processing history, and temperature. This work presents an experimental methodology for the electrical resistance-based characterization of AISI 1045 carbon steel specimens using controlled current injection and parameter estimation. The proposed system integrates a DC-modulated excitation stage, a four-terminal measurement configuration, voltage, current, and temperature acquisition, signal preprocessing, and offline resistance estimation using Python 3 scripts. Ten cylindrical specimens were evaluated through 10 repeated characterization experiments per sample, yielding 100 characterization records, each containing 100,000 voltage, current, and temperature measurements. The sample resistance is estimated using a least-squares formulation based on synchronized voltage–current data and is compared with a mean-based estimator derived from instantaneous resistance values. The results show that the least-squares estimator produced repeatable resistance values concentrated within a narrow interval, from approximately 443.43μΩ to 445.55μΩ, with interquartile ranges below 4.46μΩ for all specimens. In contrast, the mean-based estimator exhibited larger resistance values and substantially higher dispersion. The least-squares resistance-derived resistivity values ranged from $1.823\times 10^{-7}$ to $2.263\times 10^{-7}\;\Omega \;\cdot \;$m, which is consistent with the expected order of magnitude for AISI 1045 steel when compared with temperature-dependent theoretical reference values. The corresponding relative differences ranged from 1.94% to 11.00%, with most specimens remaining below 6%. These findings indicate that controlled current injection, combined with four-terminal sensing, signal preprocessing, and least-squares estimation, provides a reproducible framework for the low-resistance characterization of carbon steel specimens. Although the present study focuses on AISI 1045 steel, the methodology may be adapted to other metallic specimens, steel grades, heat-treated samples, or controlled thermal-cycle experiments, provided that geometry, temperature, calibration, and material-specific reference conditions are properly considered.

## 1. Introduction

Carbon steels constitute a major fraction of steel products used in structural and industrial applications [1,2,3]. Therefore, quality assurance frequently relies on monitoring measurable properties that correlate with performance. Mechanical metrics (e.g., strength and hardness) are routinely used for acceptance and process control; however, electrical properties can serve as complementary, indirect indicators of composition, microstructure, and processing conditions, and may provide greater sensitivity to subtle material variations that are not readily detected by bulk mechanical metrics alone [4,5,6].

Among electrical properties, electrical resistance is particularly noteworthy for its ease of monitoring via relatively straightforward current–voltage measurements and its applicability in conjunction with mechanical or thermal experimental setups [4,7,8]. For example, Ferdiansyah et al. [9] conducted measurements of the electrical resistance of steel-fiber-reinforced material utilizing a two-probe current-injection and voltage measurement methodology, and employed the resistance response to analyze the material under compressive load.

In prior work, fatigue damage and deformation in steel have been investigated using real-time electrical resistance monitoring with four-terminal measurements, in which a controlled current is applied, and the resulting potential difference is measured with high-accuracy instrumentation [10]. Related approaches have also been reported for steel specimens under tensile loading, using current-injection and voltage-sensing instrumentation to monitor changes in resistance during mechanical tests [4,11].

Among the factors influencing steel performance, the carbon-to-alloying-element ratio plays a critical role in determining final properties. Even minor variations in carbon concentration can significantly affect mechanical characteristics such as strength, hardness, and ductility [12,13,14]. In addition, electrical transport properties offer supplementary insights: in steels, resistivity is influenced by composition and microstructural state and is affected by thermal processing, which justifies its application as a non-destructive indicator of processing history and material condition [14,15,16]. This context motivates the use of electrical resistance measurements to characterize and differentiate carbon steels.

To support subsequent characterization of carbon steels, an experimental data acquisition method has been developed to estimate electrical resistance and derive resistivity from the specimen geometry. Resistance estimations are performed using a four-terminal configuration suited for low-resistance specimens, reducing the influence of lead and contact resistances [17,18,19,20]. The acquired current–voltage data are then used to estimate specimen resistance and to support subsequent resistivity-based analysis during characterization.

This paper introduces a measurement and estimation methodology for AISI 1045 carbon steel specimens that integrates chopper-modulated current excitation, four-terminal voltage sensing, and least-squares estimation based on synchronized current–voltage data. The proposed framework provides a reproducible approach for estimating electrical resistance and deriving electrical resistivity in low-resistance steel specimens under controlled experimental conditions. By combining controlled excitation with a system-identification-based estimator, this work establishes a methodological basis for future studies to correlate electrical resistance and resistivity with material composition, processing history, and microstructural state. Consequently, the approach may support subsequent developments in steel condition assessment, AISI grade differentiation, and analysis of changes in resistance associated with thermal or phase-transformation processes.

## 2. Materials and Methods

This section details the steel specimens used, the relevant electrical properties, the devices used, and the measurement methodology implemented to estimate the electrical resistance of very-low-value steel specimens. Particular attention is given to the definitions of electrical resistance and resistivity, as well as the four-point measurement configuration. This configuration has been selected to minimize systematic errors related to lead and contact resistances. Next, the Sinusoidal Pulse Width Modulation (SPWM) technique is introduced as the experimental excitation strategy selected for generating controlled current and voltage variations. This input design is chosen to meet system-identification principles by delivering informative, repeatable dynamics that enhance the robustness of resistance estimation.

### 2.1. Specimens’ Electrical Resistance

Cylindrical specimens were machined from a round bar of AISI 1045 steel with a chemical composition of Fe–0.46C–0.26Si–0.60Mn (wt.%). The steel was considered in the as-wrought condition, which is commonly associated with a ferritic–pearlitic microstructure in medium-carbon steels. AISI 1045 steel was selected as the test material because it is a widely used medium-carbon steel with well-documented mechanical, thermal, and microstructural behavior. Previous studies report its use in several industrial components, where strength, wear resistance, machinability, and heat-treatment response are relevant design requirements [21,22]. In this context, AISI 1045 serves as a suitable reference material to validate the proposed electrical resistance estimation methodology before extending the approach to other steel grades.

**Figure 1:**
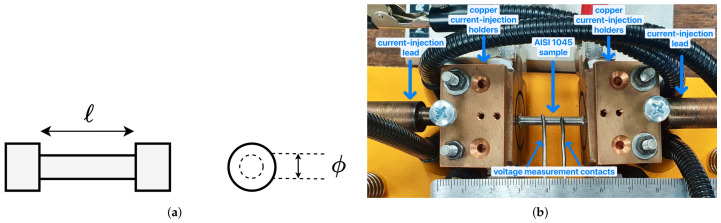
AISI 1045 carbon steel specimen and experimental measurement configuration. (**a**) Mechanical schematic of the specimen geometry, indicating the effective length *ℓ* and diameter ϕ. (**b**) Four-terminal experimental setup for the sample, showing the copper current-injection holders and central voltage-sensing probes; a ruler is included to provide a visual scale reference.

Each specimen had a nominal length *ℓ* = 26 mm and a diameter ϕ = 4 mm, as illustrated in the mechanical drawing in Figure 1a. The geometric dimensions required for resistivity calculations were measured for each specimen and used in subsequent analyses. Figure 1b shows the experimental arrangement, including the sample, current-injection contacts, and voltage-sensing points used for the four-terminal resistance measurements.

The electrical resistance is a general and macro measurement that relates the ratio between the measured potential difference *u* across the specimen and the applied current *i*, under the assumption that the specimen behaves predominantly as an ohmic element within the selected excitation range; therefore, R≈u/i. This definition underlies four-terminal resistance measurements in conductive materials [4,17,23].

However, electrical conduction in metals is primarily supported by free electrons that drift under an applied electric field while undergoing frequent collisions with the lattice. These scattering events lead to energy dissipation as Joule heating, which can modify the electrical resistance through the temperature dependence of the resistivity [18,24,25]. Therefore, the specimen temperature T(t) is measured together with the voltage u(t) and current i(t) to verify quasi-stationary conditions within each estimation experiment.

At the macroscopic scale and for approximately constant temperature, metallic conduction is commonly modeled by a linear relationship between current density and electric field, J=σE, where σ is the electrical conductivity and ρ=1/σ is the resistivity. Under this assumption, the specimen exhibits an approximately linear voltage–current relation, supporting the estimator model u(t)=R·i(t)+e(t) adopted in this work, where e(t) denotes measurement noise and unmodeled effects in the voltage measurement.

To assess measurement consistency, the linear relationship between the measured signals is evaluated by calculating the Pearson correlation coefficient between u(t) and i(t) over each acquisition window using Equation (1):

(1) $$r_{ui}=\frac{\sum_{t=1}^{N}\left(u\left(t\right)-\bar{u}\right)\left(i\left(t\right)-\bar{i}\right)}{\sqrt{\sum_{t=1}^{N}{\left(u\left(t\right)-\bar{u}\right)}^{2}}\sqrt{\sum_{t=1}^{N}{\left(i\left(t\right)-\bar{i}\right)}^{2}}}$$

where $\bar{u}$ and $\bar{i}$ are the mean voltage and mean current, respectively, calculated over the acquisition window, *N* is the number of samples —measurements— in the window, and *t* is the sample index.

The voltage–current correlation coefficient $r_{ui}$ was used to assess the consistency of the acquired measurements. High values of $r_{ui}$ indicate that the voltage and current signals exhibit an approximately linear relationship within the analyzed measurement data, which is consistent with an approximately ohmic response of the specimen under the applied excitation. After the experiments were completed, the acquired voltage and current data were used to estimate the sample’s electrical resistance. The resistance-estimation methods used for this purpose are introduced in the following subsections. The resulting resistance estimates, denoted as $\hat{R}$, were then converted into electrical resistivity, represented by ρ using the measured specimen geometry in accordance with Equation (2).

(2) $$\rho =\hat{R}\frac{A}{\ell },$$

where $\hat{R}$ is the estimated specimen resistance in Ω, ρ is the electrical resistivity in Ω·m, *ℓ* is the specimen length in m, and *A* is the cross-sectional area in $m^{2}$.

### 2.2. Four-Terminal Resistance Estimator

The four-terminal method (Kelvin connection) employs separate terminals for current injection (force) and voltage sensing (sense), so that the measured potential difference is taken across a defined gauge length of the specimen while minimizing the influence of lead, contact, and connector resistances. This configuration is widely used for low-resistance measurements and is historically associated with Kelvin bridge techniques developed for accurate resistance metrology.

**Figure 2:**
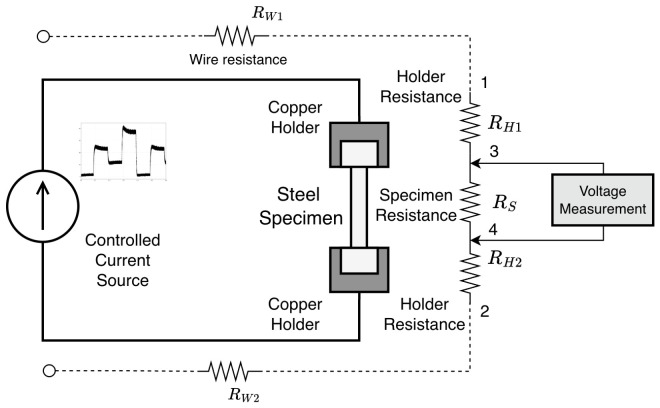
Four-terminal measurement model used for the electrical resistance estimation of the AISI 1045 steel specimen. The controlled current source forces current through the system circuit, including the wire resistances $R_{W1}$ and $R_{W2}$, the copper holders $R_{H1}$/$R_{H2}$, and the specimen $R_{S}$. The equivalent model separates the holder resistances $R_{H1}$ and $R_{H2}$ from the specimen resistance $R_{S}$. The voltage is measured through separate sensing terminals connected across the specimen segment.

In this work, the Kelvin configuration is combined with system-identification concepts by applying a designed excitation current to the specimen–holder system and recording the corresponding voltage response across the gauge length. The resulting current–voltage data provide the necessary input–output record for the resistance estimator $\hat{R}$, while the excitation enhances numerical conditioning and reduces bias attributable to measurement noise and non-ideal contacts. Therefore, the general idea of the four-terminal resistance estimator setup and the main input–output signals—current *i* and voltage *u*—are depicted in Figure 2.

As shown in Figure 2, the outer circuit represents a simplified electrical model of the resistance contributions present in the identification system. The applied current flows through the connection leads (1 and 2), copper holders (contact interfaces), and the steel specimen, which are represented by $R_{W1}$, $R_{W2}$, $R_{H1}$, $R_{H2}$, and $R_{S}$, respectively. Thus, the sample’s voltage is measured by directly sensing the terminals (3 and 4) connected across the specimen sample $R_{S}$.

### 2.3. Least-Squares Resistance Estimator

Considering the measured input current *i* and the output voltage *u*, sampled at discrete instants *n* (i.e., $i_{n}$ and $u_{n}$ denote the *n*-th samples acquired at time index *n*), a least-squares estimator (LSE) is formulated by minimizing the mean squared error (MSE) [26]. Equation (3) defines the residuals as the difference between each measured sample $u_{n}$ and the model prediction ${\hat{u}}_{n}=R\;\cdot \;i_{n}$. The MSE cost function is then obtained by averaging the squared residuals over *m* measured instances. Setting the derivative of this cost function with respect to *R* to zero yields the closed-form estimate of $\hat{R}$. Equations (3)–(7) detail this derivation, including substitution of ${\hat{u}}_{n}$, differentiation, and solving for $\hat{R}$.

(3) $$\begin{array}{cc} MSE & =\frac{1}{m}\sum_{n=1}^{m}{\left(u_{n}-{\hat{u}}_{n}\right)}^{2} \end{array}$$

(4) $$\begin{array}{cc} MSE(R) & =\frac{1}{m}\sum_{n=1}^{m}{\left(u_{n}-R\;\cdot \;i_{n}\right)}^{2} \end{array}$$

(5) $$\begin{array}{cc} \frac{𝜕\;MSE(R)}{𝜕R} & =\frac{2}{m}\sum_{n=1}^{m}\left(u_{n}-R\;\cdot \;i_{n}\right)\left(-i_{n}\right) \end{array}$$

(6) $$\begin{array}{cc} 0 & =\sum_{n=1}^{m}\left(-u_{n}i_{n}\right)+\sum_{n=1}^{m}\left(R\;\cdot \;i_{n}^{2}\right) \end{array}$$

(7) $$\begin{array}{cc} \hat{R} & =\frac{\sum_{n=1}^{m}u_{n}i_{n}}{\sum_{n=1}^{m}i_{n}^{2}} \end{array}$$

where

- $\hat{R}$: Least-squares estimate of *R*.
- *m*: Number of sampled data points instances.
- *n*: Discrete-time sample index (n=1,2,…,m).
- $u_{n}$: Measured voltage at sample *n*.
- $i_{n}$: Measured current at sample *n*.

### 2.4. Experimental Setup

**Figure 3:**
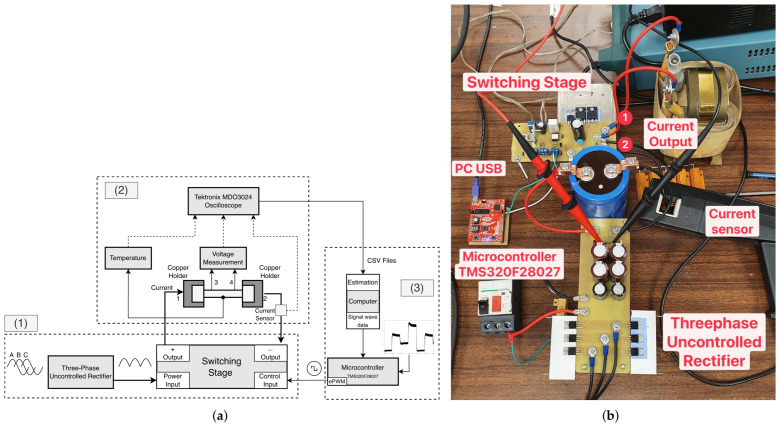
Experimental setup used for the SPWM-based excitation and measurement of current, voltage, and temperature in the steel specimens. (**a**) Schematic representation of the complete setup, including (**1**) the power stage: three-phase uncontrolled rectifier and switching stage; (**2**) the measurement stage: current, voltage, and temperature acquisition; and (**3**) the logging and control stage. (**b**) Photograph of the implemented experimental platform, showing the three-phase uncontrolled rectifier, SPWM switching stage, current-output terminals, current sensor, PC-USB interface, and the TMS320F28027 microcontroller used for control and signal generation.

Figure 3 illustrates the comprehensive block diagram of the implemented experimental setup. It consists of three primary components: (1) the power stage, (2) the measurement stage, and (3) the logging and control stage.

#### 2.4.1. Power Stage

The power stage, shown in Figure 3(a1), was designed to supply a controlled current excitation to the steel specimen during the four-terminal resistance measurements. It consists of a three-phase full-wave uncontrolled rectifier supplied by the three AC input phases, denoted as A, B, and C, followed by a DC-link capacitor bank and a chopper-based switching stage. The rectifier was implemented using six BYV32-200 ultrafast rectifier diodes (Vishay Intertechnology, Malvern, PA, USA) and provided an open-circuit DC output voltage of approximately 14 V. An 116,640 μF capacitor bank was connected to the DC link to reduce voltage ripple and voltage sags caused by the chopper stage’s current demand. The main components of the implemented power stage are identified in Figure 3b.

The switching stage was implemented utilizing an IRF3205 N-channel power MOSFET (International Rectifier, El Segundo, CA, USA), its corresponding gate driver, two HFA30PA60C freewheeling diodes (Vishay Intertechnology, Malvern, PA, USA) connected in parallel, and a 64.5 *H* output inductor. The steel specimen was mounted at the switching stage output employing two-part copper holders, which provided the high-current pathway through the sample. This switching stage modulates the DC bus in accordance with the control input, while the output inductance contributes to obtaining a continuous current waveform across the specimen. During the experiments, the operating range was selected to maintain continuous conduction and limit the maximum current to approximately 40 A, thereby protecting the power semiconductors and ensuring consistent excitation conditions. Consequently, the power stage functioned as a controlled current excitation source for the resistance estimator.

#### 2.4.2. Measurement Stage

The measurement stage, as depicted in Figure 3(a2), includes the temperature, current, and voltage sensors. The numbers adjacent to the steel sample denote the current-injection leads —1 and 2— and the voltage measurement points —3 and 4—.

The specimen is mounted in a two-part conductive copper holder that provides a stable electrical contact for current injection; see Figure 1b and Figure 3(a2). The holder is manufactured from high-conductivity copper to minimize parasitic effects at the current contacts, while the voltage is measured using separate terminals across the specified sample length, as required by the four-terminal setup.

Because the measured voltage is in the millivolt range and is affected by high-frequency components associated with the switching stage commutation and environmental electrical noise, the signal is processed using a custom-designed second-order low-pass filter circuit. This filter attenuates unwanted noise components and amplifies the voltage signal prior to acquisition, thereby improving the effective resolution of the measured voltage. In the post-processing stage, the amplification factor is compensated, and the filter-induced delay is corrected by the later processing script. This delay was measured as (Δt=3.2ms); considering a sampling period of 20μs, the voltage signal was shifted by 160 samples to improve synchronization with the current record before applying the least-squares resistance estimator.

The excitation current is measured using a TCP0150 AC/DC current probe (Tektronix, Inc., Beaverton, OR, USA) with a nominal range of 0–150 A, clamped directly onto the copper holder conductor. Temperature is monitored using an OPTCTLLTSF CTlaser LT infrared temperature module (Optris GmbH, Berlin, Germany), which delivers a 0–5 V analog output corresponding to a 0–500 °C measurement range, with a sensitivity of 10 mV/°C. The current, voltage, and temperature-related analog signals are acquired using a Tektronix MDO3024 mixed-domain oscilloscope (Tektronix, Inc., Beaverton, OR, USA). The signals are sampled every 20μs and exported in CSV format for subsequent processing and resistance estimation.

#### 2.4.3. Control Stage and Logging

The control and data-logging stage, shown in Figure 3(a3), generates the main signal applied to the MOSFET in the DC–DC switching circuit and records the electrical and temperature measurements during each experiment. The control signal is implemented as a pulse-width-modulated (PWM) waveform generated by the enhanced PWM module of a TMS320F28027 microcontroller. The duty-cycle values employed by the PWM module are precalculated as discrete arrays and subsequently loaded into the microcontroller, utilizing the Sinusoidal Pulse Width Modulation (SPWM) technique implemented in a Python 3 script.

**Figure 4:**
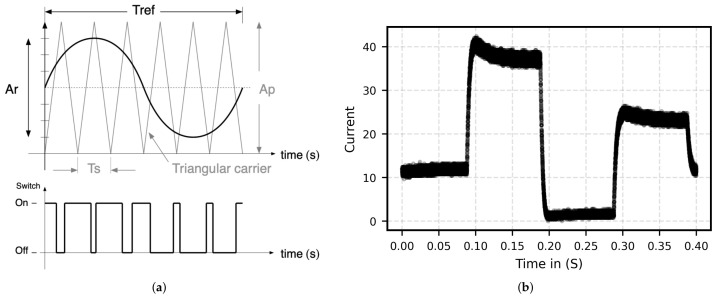
Excitation strategy approach for the control stage. (**a**) Schematic representation of the SPWM principle, in which the comparison between the reference signal—sinusoidal—and the triangular carrier defines the pulse-width sequence used to drive the MOSFET switch. (**b**) Example of the measured current response produced by the programmed four-step reference signal during a 0.4 s excitation window, which includes the random variation that was programmed and measured at each point.

In SPWM, a low-frequency reference sinusoidal signal is compared with a high-frequency triangular carrier signal. The switching state is determined by the relative magnitudes of the two signals: when the reference signal exceeds the carrier signal, the switch remains active; otherwise, it is turned off. See Figure 4a. Therefore, the comparison between the two discrete signals determines the pulse width within each carrier period. The resulting duty-cycle ratios are stored in a row-vector array and later loaded into the microcontroller to update the PWM module during operation.

For the reported experimental implementation, the carrier signal is a triangular waveform with a frequency of $f_{car}=5$ kHz. The desired excitation signal—reference—is programmed as a four-step waveform with $f_{ref}=2.5$ Hz or $T_{ref}=0.4$ seconds period. This reference signal is generated from 2000 random points arranged to form the four-step waveform over the 0.4 s window. The resulting sequence includes controlled random variations around a prescribed mean reference value. This preserves the mean current expected from an equivalent PWM excitation with a constant duty cycle, while introducing sufficient amplitude variation to improve the correlation between the programmed excitation and the measured voltage–current response. The 0.4 s excitation window is repeated five times, producing the complete 2 s reference signal used during each experiment; see Figure 4b.

During each experimental procedure, the current, voltage, and temperature signals are logged simultaneously using the Tektronix MDO3024 oscilloscope, as previously indicated. The oscilloscope records the signals with a sampling period of 20μs and a record length of 100,000 data points per variable. The data are exported directly as CSV files for offline processing. The experimental campaign consists of 10 AISI 1045 steel samples, with 10 repetitions performed for each sample. In total, 100 CSV files are generated. The files are stored locally in separate folders for each specimen and later processed in Python. The recorded CSV files are processed in the Jupyter Notebook interface based on Python 3. The processing includes dataset concatenation, phase-shift correction, scaling compensation, and conversion of the temperature signal. The resulting synchronized signals are then used to estimate the specimen resistance through the least-squares formulation.

## 3. Results

This section presents the experimental results obtained from 10 AISI 1045 carbon steel specimens. Each specimen is characterized by 10 repeated excitation experiments, yielding a total of 100 individual electrical characterizations. For each characterization, 100,000 synchronized measurements of voltage, current, and temperature are acquired, providing the complete dataset used for resistance estimation and statistical analysis.

Initially, representative voltage, current, and temperature signals are provided to depict the behavior of the controlled excitation current as well as the corresponding measured voltage and current responses. Subsequently, direct point-by-point resistance estimates are examined to demonstrate the dispersion introduced by instantaneous voltage–current ratios, especially in measurements affected by noise and small voltage variations. Lastly, the least-squares resistance estimator is evaluated as an alternative method for estimating resistance using voltage–current data. In this analysis, randomly selected subsets of the complete dataset are used to determine the amount of data required for the least-squares estimates to stabilize. This facilitates the examination of the robustness and data efficiency of the estimation procedure under limited data conditions.

### 3.1. Measured Signals

**Figure 5:**
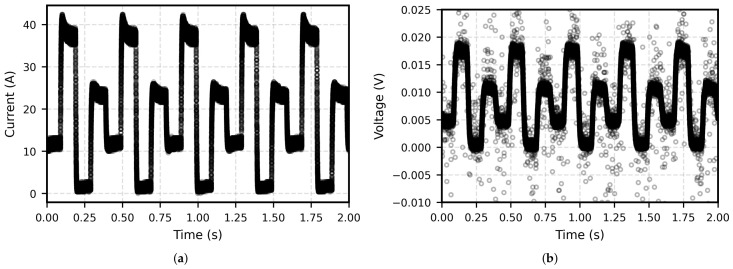
Representative experimental acquisition: (**a**) measured current signal under random-step excitation, and (**b**) measured specimen voltage under random-step excitation.

Figure 5 shows a representative acquisition obtained from one of the experimental records after signal preprocessing. In particular, the current-shift correction was applied to compensate for temporal misalignment between voltage and current measurements before estimating resistance.

The voltage and current signals, depicted in Figure 5a,b, respectively, exhibit the expected random-step behavior characterized by the controlled excitation stage. Although the voltage magnitude is small, the waveform preserves the same temporal structure as the injected current, consistent with the approximately ohmic model assumed for the specimen.

**Figure 6:**
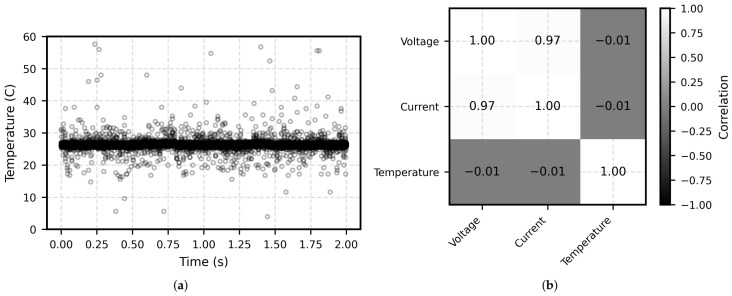
Temperature behavior and correlation matrix for a representative experimental acquisition. (**a**) Temperature signal during the 2 s acquisition window. (**b**) Correlation matrix of the preprocessed voltage, current, and temperature signals logged during the 2 s window.

Figure 6a illustrates that the temperature values remained within a narrow range during the acquisition window. This observation is pertinent because the resistance of metallic specimens is temperature-dependent; consequently, limited temperature variation supports the application of a quasi-stationary resistance model within each estimation experiment.

On the other hand, the correlation matrix shown in Figure 6b was computed from the preprocessed voltage, current, and temperature signals. The voltage and current measurements exhibit a strong positive correlation of r=0.97, supporting the use of the ohmic linear model adopted for the resistance estimator. In contrast, the correlation between temperature and the electrical variables is almost negligible, at approximately r=−0.01. This means that temperature fluctuations during the experiment were too small to produce a meaningful change in the specimen’s resistivity. Consequently, the measured voltage–current response can be attributed primarily to the imposed electrical excitation and the specimen resistance.

### 3.2. Point-by-Point Resistance Estimation and Least-Squares Convergence

**Figure 7:**
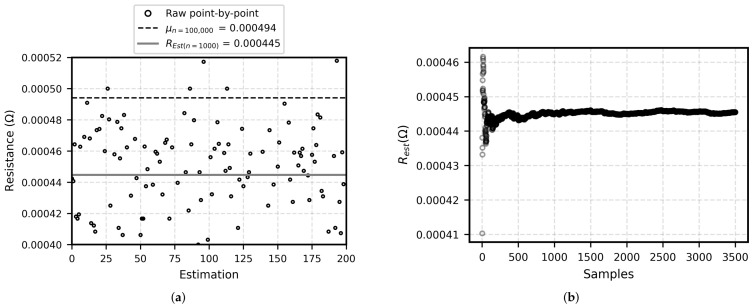
Resistance estimation from a representative experiment. (**a**) Raw point-by-point resistance values obtained from the instantaneous voltage-to-current ratio for the first 200 samples, showing the dispersion of the direct estimation and its comparison with the mean-based estimate $R_{\mu }$ computed from 100,000 points and the least-squares estimate $R_{\mathrm{Est}}$ computed from 1000 randomly selected points. (**b**) Evolution of the least-squares resistance estimate $R_{\mathrm{Est}}$ as a function of the number of randomly selected samples, showing the initial variability and the subsequent stabilization of the estimated resistance value.

An initial raw point-by-point resistance estimation is computed from the instantaneous ratio between the voltage and current samples; see the first 200 estimations in Figure 7a marked with circle points. This direct estimation exhibits considerable dispersion. This behavior is expected because the voltage signal is in the millivolt range and is affected by electrical noise, residual phase misalignment, and uncertainty in the conditioning stage. The plot also includes the resistance values obtained using a mean-based estimator ($R_{\mu }$) and the proposed least-squares estimator ($R_{\mathrm{Est}}$). The mean-based estimator $R_{\mu }$ uses all 100,000 points per experiment, whereas $R_{\mathrm{Est}}$ is computed using only 1000 randomly sampled voltage–current points.

Prior to determining the number of samples used in the least-squares estimation, an initial convergence analysis was conducted to assess the quantity of randomly selected voltage–current points necessary for obtaining a stable resistance estimate. Figure 7b depicts the progression of the estimated resistance as the number of samples increases. Following an initial transient phase, the estimator converges towards a stable value, indicating that a limited subset of randomly selected samples is adequate to reduce sample-specific fluctuations and produce a consistent resistance estimate. Consequently, the least-squares estimator utilizes a set of 1000 random samples to derive a single resistance estimate according to Equation (7).

### 3.3. Comparison Between Least-Squares and Mean-Based Estimators

The complete data processing and resistance estimation procedure is implemented using Python version 3.13. The general script is outlined in Algorithm 1 and discussed next. The algorithm imports CSV files generated by the oscilloscope during each characterization experiment, stores the acquired signals—voltage, current, and temperature—in a DataFrame variable, and performs the necessary preprocessing to synchronize the signals and correct the scaling. After preprocessing, the script evaluates each acquisition record and computes two resistance estimators: $R_{\mu }$, obtained from the mean of the instantaneous voltage–current resistance values, and $R_{\mathrm{Est}}$, obtained from the least-squares formulation. The procedure is repeated 10 times for each of the 10 steel specimens, yielding a total of 100 processed characterization records. These estimated values are subsequently utilized to generate the box-plot comparisons and a results comparison table.

**Figure 8:**
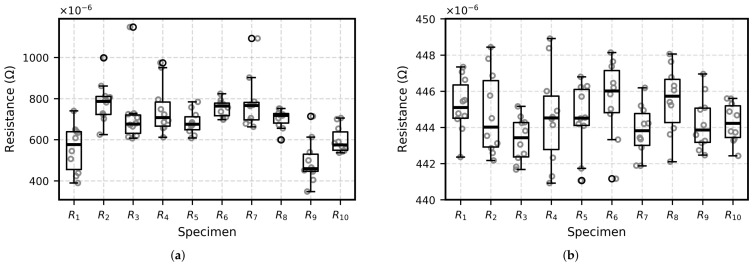
Comparison of resistance estimates for the 10 AISI 1045 specimens: (**a**) mean-based estimator obtained from instantaneous resistance values, and (**b**) proposed least-squares resistance estimator using 1000 randomly selected points per acquisition. Each box plot summarizes 10 repeated acquisition experiments per specimen, with gray dots indicating the individual estimates.

Figure 8 compares the 100 resistance estimates obtained for the 10 specimens using the two estimation approaches. Figure 8a shows the results of the mean-based estimator computed from the instantaneous resistance values. The estimates are in the order of $10^{-6}\;\Omega$, as indicated by the axis scale. Although the median resistance values remain within a similar micro-ohm range for most specimens, the box plots reveal considerable dispersion among repeated experiments over each sample. This behavior is particularly evident for specimens such as $R_{2}$, $R_{3}$, $R_{4}$, and $R_{7}$, where the interquartile ranges and whiskers are wider, and several outliers are indicated. In addition, specimens such as $R_{9}$ show lower median resistance values but still exhibit noticeable variability. These results indicate that the mean-based estimator is sensitive to sample-wise fluctuations, low-current regions, and measurement noise, which can produce unstable resistance estimates when instantaneous ratios are averaged directly.

On the other hand, Figure 8b shows the resistance values obtained with the proposed least-squares estimator using 1000 randomly selected points per characterization experiment. In contrast to the mean-based estimator, the estimated values are concentrated within a narrow interval, approximately from $440\times 10^{-6}\;\Omega$ to $450\times 10^{-6}\;\Omega$. The interquartile ranges are substantially smaller for most specimens, and the medians remain close to the central resistance region, mainly between $443\times 10^{-6}\;\Omega$ and $446\times 10^{-6}\;\Omega$. Although some outliers are still present, their effect is limited compared with the dispersion observed in Figure 8a. These results indicate that the least-squares estimator reduces the influence of sample-wise fluctuations and provides more repeatable resistance estimates under the proposed excitation and acquisition methodology.    
**Algorithm 1**: Data Processing and Resistance Estimation
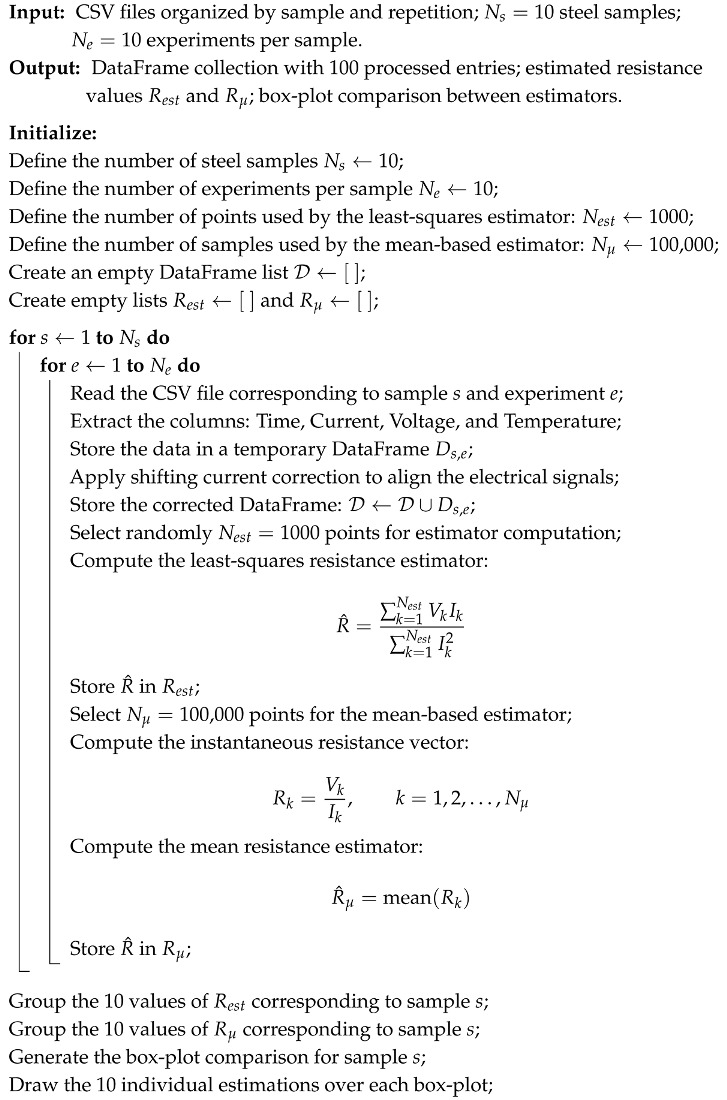


### 3.4. Theoretical and Experimental Resistivity

The theoretical resistivity is estimated using a linear approximation of the temperature-dependent AISI 1045 resistivity data reported as a material property for finite-element simulation by Gao et al. [27]:

(8) $$\rho \left(T\right)=6.875\times 10^{-10}T_{AVG}+1.8625\times 10^{-7}$$

For an average temperature close to 27°C,

(9) $$\rho_{th}\approx 2.05\times 10^{-7}\;\Omega \;\cdot \;m,$$

This theoretically computed resistivity value $\rho_{th}$ aligns closely with the values documented in the literature for AISI 1045 steel. Quiroga Agurto et al. [28] reported values ranging from approximately $1.64\times 10^{-7}$ to $2.51\times 10^{-7}\;\Omega \;\cdot \;m$, corresponding to different heat-treated samples. Similar values are also documented by Camaraza-Medina et al. [29], who compiled average resistivity values of $1.7\times 10^{-7}$ and $2.2\times 10^{-7}\;\Omega \;\cdot \;m$ at 0 °C and 100 °C, respectively.

Next, the experimental resistivity of the 10 samples evaluated in this work is computed from the least-squares median estimated resistance and the measured geometry of each specimen using Equation (10):

(10) $$\rho_{Est}={\tilde{R}}_{Est}\frac{A}{\ell },\;A=\frac{\pi \phi^{2}}{4}$$

where ${\tilde{R}}_{\mathrm{Est}}$ represents the median resistance obtained from the 10 repetitions of the least-squares estimator for each specimen, *ℓ* is the measured sample length, and ϕ is the measured sample diameter. Thus, $\rho_{\mathrm{Est}}$ is computed individually for each specimen from its corresponding estimated resistance and measured geometry. The resulting values are included in Table 1.

Thus, the relative difference between the theoretical $rho_{th}$ and experimental resistivity $rho_{Est}$ values is computed individually for each specimen as

(11) $$\varepsilon_{\rho }=\left|\frac{\rho_{\mathrm{Est}}-\rho_{\mathrm{th}}}{\rho_{\mathrm{th}}}\right|\times 100,$$

where $\rho_{\mathrm{Est}}$ is the experimental resistivity computed for each sample using its measured geometry and estimated resistance, while $\rho_{\mathrm{th}}$ is the theoretical resistivity evaluated at the corresponding measured temperature *T* during each experiment. Thus, the comparison is performed on a sample-by-sample basis. The resulting relative differences are also reported in Table 1.

### 3.5. Summary of Experimental Results

Table 1 summarizes the resistance and resistivity results obtained from the 100 characterization experiments conducted on the 10 samples $R_{1}$ to $R_{10}$. For each specimen, the table reports the average temperature *T*, the measured geometry length *ℓ* and diameter ϕ, the median value of the least-squares resistance estimator ${\tilde{R}}_{\mathrm{Est}}$, its interquartile range ${\mathrm{IQR}}_{R_{\mathrm{Est}}}$, the median value of the mean-based estimator ${\tilde{R}}_{\mu }$, and its corresponding interquartile range ${\mathrm{IQR}}_{R_{\mu }}$. In addition, the electrical resistivity $\rho_{\mathrm{Est}}$ is calculated using the ${\tilde{R}}_{\mathrm{Est}}$ and the measured specimen geometry. The theoretical resistivity $\rho_{\mathrm{th}}$ and the relative difference between $\rho_{\mathrm{Est}}$ and $\rho_{\mathrm{th}}$ are also included to provide a direct comparison with the expected reference value for the material.

As shown in Table 1, the least-squares estimator produced highly consistent resistance values among the 10 samples. The median resistance values ${\tilde{R}}_{Est}$ remained within a narrow interval, from approximately 443.43μΩ to 445.55μΩ. Likewise, the interquartile ranges were small for all specimens, remaining below 4.46μΩ. These results indicate that the proposed least-squares approach provides repeatable resistance estimates under the proposed methodology.

The resistivity values calculated from the least-squares resistance estimates also showed consistent behavior among the specimens. Because the resistivity calculation incorporates the measured geometry of each specimen, small variations in length and diameter are reflected in the final $\rho_{\mathrm{Est}}$ values. The relative differences with respect to $\rho_{\mathrm{th}}$ provide an additional criterion to assess the agreement between the experimental estimation and the theoretical reference value. In general, the reported differences indicate that the proposed methodology yields resistivity estimates within a consistent range for the tested AISI 1045 carbon steel specimens.

By contrast, the mean-based estimator yielded larger resistance values and substantially higher dispersion. The corresponding median values ranged from approximately 616.60μΩ to 725.13μΩ, while the interquartile ranges reached values above 200μΩ in some specimens. This behavior indicates that the mean-based estimator is more susceptible to sample-wise fluctuations, measurement noise, and low-current regions. Therefore, it does not provide the same level of repeatability as the least-squares method for the proposed experimental conditions.

## 4. Discussion

The experimental results indicate that the proposed methodology provides reproducible estimates of very low electrical resistance for AISI 1045 steel specimens under controlled current injection. The box plot and analysis show that the dispersion of the estimated resistance values remains limited across repeated experiments, supporting the repeatability of the measurement and post-processing procedures. These results are pertinent because the voltage measurements are within the millivolt range, where electrical noise, signal-conditioning effects, and residual phase misalignment can substantially impact standard resistance calculations. In this context, the least-squares estimator, combined with randomly selected points, mitigates the influence of measurement fluctuations by leveraging the underlying relationship between the measured voltage and current signals.

The comparison of the hundred $R_{\mu }$ and $R_{Est}$ also highlights the methodological advantage of using the proposed model-based estimator over relying solely on direct point-by-point or average-based resistance estimations. Although $R_{\mu }$ employs a larger sample size—100,000—$R_{Est}$ offers a more concise estimate—1000 random points—derived from the voltage–current correlation. It is less susceptible to transient fluctuations resulting from noise or low-significance excitation values. Therefore, the findings indicate that integrating four-terminal sensing, controlled-current excitation, and least-squares estimation with random sample points is appropriate for low-resistance measurements in studies of metallic specimens.

From a broader perspective, the proposed methodology can be extended to the analysis of other steels, provided that the experimental conditions, specimen geometry, temperature range, and calibration procedure are properly adapted to the material under study. Electrical resistance and resistivity are sensitive to composition, microstructure, processing history, and thermal state; therefore, different steel grades would require independent validation and reference measurements before quantitative comparisons can be established. Nevertheless, the general methodology proposed in this work is not restricted to AISI 1045 steel. Its main contribution lies in integrating controlled-current excitation with parameter-estimation principles designed for low-resistance characterization. In this context, the random-step current waveform improves the excitation of the specimen–holder system, while the post-processing stage reduces the influence of instrumentation noise, residual phase misalignment, and signal-conditioning effects. Consequently, the method provides a practical and reproducible basis for estimating low electrical resistance values in steel specimens and could be adapted for comparative studies involving other carbon steels, alloy steels, or thermally processed metallic samples.

The main limitation of the current study is that resistance is estimated as an offline post-processing step. In addition, the experiments were conducted within a limited temperature range, so the influence of temperature-dependent resistivity, thermal expansion, oxidation, and microstructural transformations was not fully assessed. Future work should consider online implementation of the estimator, improved synchronization between voltage and current channels, temperature-controlled experiments, and long-duration heat treatments. These extensions would enable the method to be evaluated for real-time monitoring of changes in electrical properties during thermal processing and for constructing datasets for material classification, process-quality assessment, or material characterization.

## 5. Conclusions

This work presented an experimental methodology for estimating the low electrical resistance and resistivity of AISI 1045 carbon-steel specimens using controlled-current excitation, four-terminal voltage sensing, signal preprocessing, and least-squares parameter estimation. The proposed approach was evaluated using 100 characterization experiments on 10 steel specimens, with voltage, current, and temperature measurements acquired under controlled excitation conditions.

The results showed that the least-squares estimator yielded repeatable resistance estimates despite the millivolt-level voltage response and measurement noise. The estimated resistance values obtained with ${\tilde{R}}_{\mathrm{Est}}$ were concentrated within a narrow interval, from approximately 443.43μΩ to 445.55μΩ. In addition, the interquartile range remained below 4.46μΩ for all specimens. This behavior contrasts with the larger dispersion observed in direct point-by-point resistance calculations, indicating that the proposed estimator reduces the influence of sample-level fluctuations, low-current regions, residual phase misalignment, and signal-conditioning noise.

The correlation analysis supported the validity of using an ohmic model during the analyzed data. After the shifting correction, the voltage and current signals exhibited a strong positive correlation, confirming that the measured voltage response is primarily attributable to the imposed current excitation. In contrast, the temperature remained nearly constant during each experiment, so temperature-induced variations in resistivity were not the dominant factor in the estimated values. These results indicate that, under the selected experimental conditions, the resistance can be treated as quasi-stationary during the estimation process.

The physical consistency of the estimated resistance values was further assessed through the corresponding electrical resistivity. Using the measured sample geometry, the resistivity obtained ranged from $1.823\times 10^{-7}$ to $2.263\times 10^{-7}\;\Omega \;\cdot \;m$ for the 10 AISI 1045 steel samples. These values were compared with a previous reported resistivity model $\rho_{\mathrm{th}}$ evaluated at the measured temperature of each specimen, which ranged from $2.041\times 10^{-7}$ to $2.064\times 10^{-7}\;\Omega \;\cdot \;m$. The corresponding relative differences ranged from 1.94% to 11.00%, with most specimens remaining below 6%. These results indicate that the proposed methodology provides resistance-derived resistivity values within the expected order of magnitude for reported AISI 1045 steel. Therefore, the estimator is not only numerically repeatable but also physically meaningful when the measured specimen geometry and temperature-dependent reference values are properly considered.

The main contribution of this study is the integration of the controlled excitation source and least-squares estimation approach into a reproducible framework for low-resistance characterization of metallic specimens. Rather than relying only on instantaneous voltage-to-current ratios, the proposed method uses a set of randomly selected measurements to estimate a single resistance value that best represents the voltage–current relationship during each experiment. This makes the method suitable for experimental conditions in which very low voltages, noise, and signal-conditioning effects limit the reliability of conventional direct calculations.

The reported results support that the proposed methodology is effective for the electrical-resistance characterization of AISI 1045 carbon-steel specimens under the selected current excitation and monitoring temperature conditions. The estimator produced repeatable resistance and resistance-derived resistivity values consistent with the expected range for this material. These findings support the use of the proposed framework methodology as a practical tool for estimating low resistance in metallic specimens, particularly when low-level voltage responses and measurement noise limit the reliability of direct point-by-point calculations. Although the experimental results focused on AISI 1045 steel, the methodology is based on general principles of four-terminal sensing, controlled excitation, signal preprocessing, and least-squares parameter estimation. Therefore, it can be extended to other steel grades, carbon-content variations, heat-treated specimens, and controlled thermal-cycle experiments, as well as future studies related to phase transformations and material-state assessment.

Future work should include validation against reference low-resistance instrumentation or methods, uncertainty analysis of the measurement chain, tests over wider temperature ranges, and comparisons among steels with different compositions and thermal histories. These extensions would allow the proposed methodology to evolve from repeatable resistance estimation toward material classification, monitoring of thermally induced transformations, and the development of datasets for data-driven characterization of carbon steels.

## Figures and Tables

**Table 1:** Comparison between the least-squares and mean-based resistance estimators for the 10 carbon-steel samples, conducted over 100 characterization experiments. The last three columns report the experimental resistivity $\rho_{\mathrm{Est}}$ computed from $\tilde{R}\mathrm{est}$ and the measured specimen geometry, the theoretical resistivity $\rho_{\mathrm{th}}$ evaluated at the measured temperature of each specimen, and the corresponding relative difference $\varepsilon_{\rho Est}$.

Sample	*T*	*ℓ*	ϕ	$\tilde{R}\mathrm{Est}$	${\mathrm{IQR}}_{R\mathrm{Est}}$	$\tilde{R}\mu$	${\mathrm{IQR}}_{R_{\mu }}$	$\rho_{\mathrm{Est}}$	$\rho_{\mathrm{th}}$	$\varepsilon_{\rho \mathrm{Est}}$
	[°C]	[m]	[m]	[μΩ]	[μΩ]	[μΩ]	[μΩ]	[$10^{-7},\;\Omega \;\cdot \;$m]	[$10^{-7},\;\Omega \;\cdot \;$m]	[%]
$R_{1}$	26.052	0.02662	0.00400	445.18	3.60	682.68	101.39	2.102	2.042	2.96
$R_{2}$	27.023	0.02610	0.00386	444.00	1.27	648.41	151.24	1.991	2.048	2.80
$R_{3}$	28.445	0.02632	0.00398	443.76	4.46	709.67	125.14	2.098	2.058	1.94
$R_{4}$	27.832	0.02648	0.00380	445.46	2.30	725.13	228.77	1.908	2.054	7.10
$R_{5}$	29.262	0.02680	0.00388	444.63	2.84	686.76	196.22	1.962	2.064	4.93
$R_{6}$	27.027	0.02632	0.00382	444.10	1.84	720.02	87.96	1.934	2.048	5.58
$R_{7}$	28.069	0.02512	0.00404	443.43	1.61	703.35	66.62	2.263	2.055	10.10
$R_{8}$	25.902	0.02614	0.00386	445.45	2.04	692.71	126.67	1.994	2.041	2.28
$R_{9}$	26.657	0.02682	0.00390	445.55	2.59	703.34	49.87	1.985	2.046	2.97
$R_{10}$	27.015	0.02622	0.00370	444.47	0.98	616.60	150.32	1.823	2.048	11.00

## Data Availability

The research data supporting the results reported in this study are publicly available through the Open Science Framework (OSF) at https://doi.org/10.17605/OSF.IO/DASM6 (accessed on 3 July 2026). The archived dataset includes the experimental measurements generated during the study and used to estimate the electrical resistance and resistivity of AISI 1045 steel specimens.
